# Comparison of Sensitivity and Quantitation between Microbead Dielectrophoresis-Based DNA Detection and Real-Time PCR

**DOI:** 10.3390/bios7040044

**Published:** 2017-09-30

**Authors:** Michihiko Nakano, Zhenhao Ding, Junya Suehiro

**Affiliations:** 1 Faculty of Information Science and Electrical Engineering, Kyushu University, Fukuoka 819-0395, Japan; suehiro@ees.kyushu-u.ac.jp; 2 Graduate School of Information Science and Electrical Engineering, Kyushu University, Fukuoka 819-0395, Japan; ding@hv.ees.kyushu-u.ac.jp

**Keywords:** DEP, dynamic range, dielectric microbeads, detection limit, impedance measurement, virus diagnosis

## Abstract

In this study, we describe a microbead-based method using dielectrophoresis (DEP) for the fast detection of DNA amplified by polymerase chain reaction (PCR). This electrical method measures the change in impedance caused by DEP-trapped microbeads to which biotinylated target DNA molecules are chemically attached. Using this method, measurements can be obtained within 20 min. Currently, real-time PCR is among the most sensitive methods available for the detection of target DNA, and is often used in the diagnosis of infectious diseases. We therefore compared the quantitation and sensitivity achieved by our method to those achieved with real-time PCR. We found that the microbead DEP-based method exhibited the same detection limit as real-time PCR, although its quantitative detection range was slightly narrower at 10–10^5^ copies/reaction compared with 10–10^7^ copies/reaction for real-time PCR. Whereas real-time PCR requires expensive and complex instruments, as well as expertise in primer design and experimental principles, our novel method is simple to use, inexpensive, and rapid. This method could potentially detect viral and other DNAs efficiently in combination with conventional PCR.

## 1. Introduction

DNA amplification is an essential step in most nucleic acid-based diagnosis methods, including those used in the diagnosis of infectious diseases. Polymerase chain reaction (PCR), involving the amplification of specific DNA sequences from single molecules using multiple enzymatic reaction cycles, is the best-known DNA amplification method, although others, including isothermal amplification methods, have also been developed [1]. In real-time PCR, which is also known as quantitative PCR (qPCR), optical methods are used to measure amplicon levels after each cycle. Real-time PCR has a broad dynamic range of 7–8 logarithmic decades, and is particularly useful for quantitative measurements [2,3,4]. Disadvantages of real-time PCR include the need for expensive reagents and optical detection apparatus, as well as the need for specialist knowledge for designing specific DNA probes. Although conventional PCR is easier and less expensive than real-time PCR, it requires a time-consuming DNA detection process, whereas real-time PCR results are obtained immediately. In conventional PCR, amplicons are generally separated by agarose gel electrophoresis before detection. Although agarose gel electrophoresis is the gold standard approach, it is relatively complicated and time-consuming. Thus, if amplicons from conventional PCR methods could be measured quantitatively and promptly, the combination of PCR and novel DNA detection methods could provide a viable alternative to real-time PCR.

**Figure 1:**
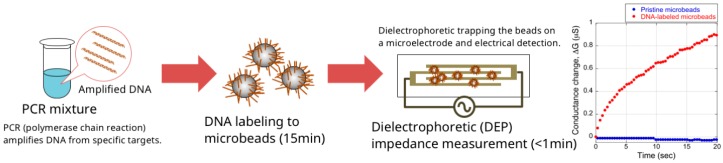
Schematic illustration of the microbead DEP-based DNA detection method.

Recently, we proposed a novel DNA amplicon detection method, using dielectric microbeads and a simple electrical setup, that was simple and able to yield results in approximately 15 min (Figure 1) [5,6]. In this method, amplicons are attached to dielectric microbeads, which have a negative dielectrophoretic (DEP) property. The DNA attachment changes the direction of the DEP force of the dielectric microbeads from negative (repulsive) to positive (attractive), because DEP is greatly affected by the surface conductance; as the DNA phosphate backbone is negatively charged, the attached DNA increases the surface conductance of the microbeads, resulting in the dynamic change of the DEP force. The DNA-labeled microbeads are then trapped on a microelectrode by positive DEP, and are detected by measuring changes in the impedance of the microelectrode (dielectrophoretic impedance measurement, DEPIM) [7,8,9].

The combination of PCR and microbead DEP-based DNA detection provides sensitive, easy, and inexpensive detection of targets. PCR can theoretically amplify specific DNA exponentially from a single target molecule. PCR also enables chemical modification of amplicons such that only specific amplicons can be attached to microbeads. The attachment of amplicons is performed by simply mixing the PCR solution and microbeads. Then, the microbead DEP enables faster DNA detection than agarose gel electrophoresis. The electrical operation, generating DEP force and measuring impedance, enables easy operation and inexpensive setup and reagents compared to fluorescent detection of DNA. Although the combination requires a longer time to detect targets than real-time PCR, this method can be performed using inexpensive microbeads and microelectrodes. It might be possible to attach amplicons to microbeads during PCR using techniques such as BEAMing [10]. If this is realized, the detection time will be similar to that of real-time PCR.

In this study, we aimed to demonstrate the quantitative detection of a viral genome using a dielectric microbead-based DNA detection method, and showed that the detection limit and dynamic range of this method are comparable to those of real-time PCR.

## 2. Theory

### 2.1. DEP of a Small Dielectric Particle

DEP is the electrokinetic motion of a dielectric material placed in a non-uniform electric field [11,12]. The DEP force (*F_DEP_*) acting on a spherical particle is given by the equation

(1) FDEP=2πr3εmRe[K(ω)]∇E2

where *r* is radius of the particle, *ε_m_* is the permittivity of the surrounding medium, *E* is the magnitude of the applied electric field, and Re[*K*(*ω*)] is the real component of the Clausius–Mossotti (CM) factor *K*(*ω*). The CM factor is given by the equation

(2) $$K\left(\omega \right)=\frac{\varepsilon_{p}^{*}-\varepsilon_{m}^{*}}{\varepsilon_{p}^{*}+2\varepsilon_{m}^{*}}$$

where ε_p_* and ε_m_* are the complex permittivities of the particle and the surrounding medium, respectively. The complex permittivity is given by the equation

(3) $$\varepsilon^{*}=\varepsilon -j\frac{\sigma }{\omega }$$

where *ε* is permittivity, *σ* is conductivity, and *ω* is the angular frequency of the applied electric field. When Re[*K*(*ω*)] is positive, the particle moves towards the high electric field region (positive DEP), and conversely, the particle moves away from this region when Re[*K*(*ω*)] is negative (negative DEP).

The conductivity of a solid dielectric particle *σ_p_* is given by the equation

(4) $$\sigma_{p}=\sigma_{b}+2\frac{K_{S}}{r}$$

where *σ_b_* and *K_S_* are the bulk conductivity and surface conductance of the particle, respectively [12,13]. In the case of dielectric particle, *σ_p_* is almost zero. Equations (1)–(4) suggest that the DEP force acting on a smaller particle should be more dependent on the surface conductance than that acting on a larger particle. *K_S_* can be divided into two distinct components: Stern layer conductance, *K_Stern_* and diffuse layer conductance, *K_Diff_* [13]. Equation (4) can be written as

(5) $$\sigma_{p}=\sigma_{b}+2\frac{K_{Stern}}{r}+2\frac{K_{Diff}}{r}$$

**Figure 2:**
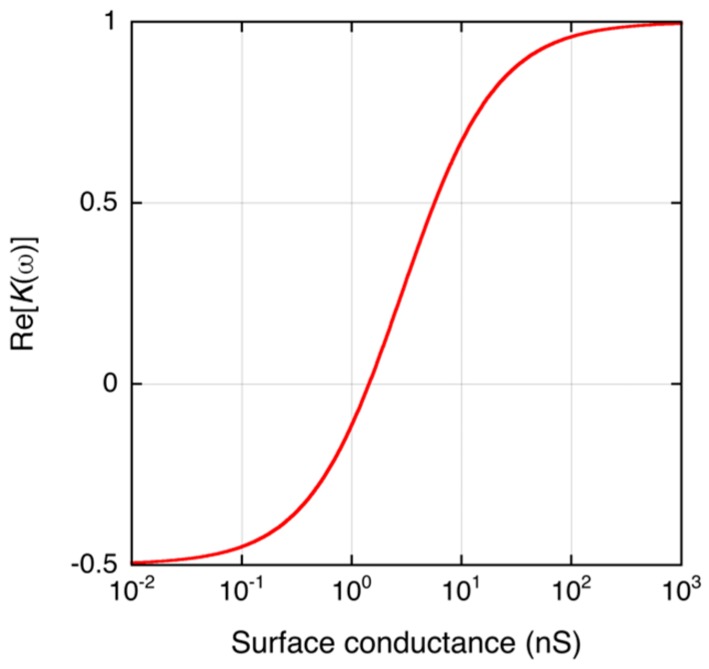
Theoretical calculation of the dependence of Re[*K*(*ω*)] of a dielectric microbead (*r* = 2.8 μm) on its surface conductance. The parameters for the calculation are: *ε_p_* = 2.4 × *ε*_0_ F/m, *ε_m_* = 78 × *ε*_0_ F/m, *ε*_0_ = 8.854 × 10^−12^ F/m, *σ_m_* = 2 × 10^−3^ S/m, and *ω* = 2π × 10^5^ rad/s. The surface conductance is typically 1 nS for latex particles [13].

The Stern layer conductance is given by the equation

(6) $$K_{Stern}=\rho_{q,Stern}\mu_{Stern}$$

where *ρ_q,Stern_* and *μ_Stern_* are the equivalent surface charge density and ion mobility in the Stern layer. Equations (5) and (6) show that if the surface charge density increases, the Stern layer conductance increases, resulting in increased conductance of the particle. When *σ_p_* increases, it results in increasing Re[*K*(*ω*)]. Figure 2 shows the relationship between *K_S_* and Re[*K*(*ω*)] of a latex microparticle calculated by Equations (2)–(4).

### 2.2. DEP of DNA-Labeled Microbeads

As surface conductance affects the DEP of the microbeads (Equations (1)–(6)), DNA immobilization thus switches the microbead DEP from negative to positive mainly because of negative charges on the DNA molecule. Generally, a double-stranded DNA molecule has two negative charges per base pair on its phosphate backbone [14]. We propose a novel detection method for DNA amplicons, utilizing the DEP of microbeads (Figure 1). Following PCR, amplicons are chemically immobilized onto the surface of microbeads having an initial negative Re[*K*(*ω*)] value, switching the DEP property to positive. The DNA-labeled microbeads are then trapped onto a microelectrode by the positive DEP, and are readily quantified by measuring the microelectrode impedance.

### 2.3. DEPIM of DNA-Labeled Microbeads

Using the DEPIM method, the impedance increase occurs when target materials are trapped by a positive DEP is measured, allowing real-time quantification of these materials [7]. If the target is dielectric, the impedance of the trapped target can be modeled as a parallel circuit of the conductance (*G*) and the capacitance (*C*). Increasing the number of trapped targets is equivalent to increasing the elements of the parallel circuit. Therefore, the number of targets can be quantified by measuring the change of *G* and/or *C*. It was previously demonstrated that DEPIM could realize quantitative detection of bacteria and viruses [8,9]. DEPIM detects the targets collected on a microelectrode under the action of positive DEP force while the target suspension flows over it. Previously published theoretical evaluations suggest that the DEPIM conductance curve could be fitted to an exponential function of elapsed time, and that the tangent slope of the curve at *t* = 0 (*t*, time) should be proportional to the target concentration [7].

In this study, the DNA-labeled microbead suspension was placed onto the microelectrode, and thus the DEP collection of targets is considered to be performance in a static solution. Such DEP collection is explained by the Fokker–Planck equation, as described in previous studies showing that the DEP collection of targets at a microelectrode also increased exponentially with time [15,16,17,18,19]. These studies show that the tangent slope of the collection curve at *t* = 0 is proportional to the magnitude of the DEP force. As predicted by the theoretical calculation shown in Figure 2, increasing the surface conductance *K_S_* changes the DEP force from negative to positive. Because DNA is negatively charged, an increasing number of the amplicons attached on the surface will increase the surface conductance due to the dependence of the surface conductance on the charge density of the surface [13]. Generally, the number of amplicons labeled on the surface is proportional to their concentration in the liquid. The tangent slope of the DEPIM curve at *t* = 0, which is proportional to the magnitude of DEP force, is thus dependent on the amplicon concentration.

## 3. Materials and Methods

Norovirus is a common cause of gastroenteritis globally, leading to outbreaks in various epidemiological settings [20]. In this study, we used a segment of the norovirus GII genome as target DNA, and created amplicons by real-time PCR using a verified norovirus detection kit. These amplicons were then used in our DEP microbead-based method.

### 3.1. Real-Time PCR

Norovirus DNA amplification and detection by real-time PCR was performed using a qPCR norovirus (GI/GII) typing kit (TaKaRa Bio Inc., Tokyo, Japan), which utilizes TaqMan probe technology [21], according to the manufacturer’s instructions. The positive control DNA included with this kit, which is derived from norovirus GII, was used as the target DNA. The primers, designed to produce a biotin-labeled 98 bp amplicon [22], were: COG2F: 5′-biotin-CAR GAR BCN ATG TTY AGR TGG ATG AG; COG2R: 5′-TCG ACG CCA TCT TCA TTC ACA (where R = A or G, and B = C, G or T). The reaction volume was adjusted to 50 μL. Real-time PCR reactions were performed using LightCycler Nano (Roche Diagnostics, Rotkreuz, Switzerland), and target DNA concentrations in the range of 10 to 10^7^ copies/reaction were examined. The reaction composition and thermal cycling (45 cycles) conditions were as recommended by the manufacturer.

### 3.2. DNA Detection Using the Microbead-Based DEPIM Method

The real-time PCR products were mixed with Dynabead M-280 magnetic microbeads (Life Technologies, Rockville, MD, USA), which have a diameter of 2.8 μm and are coated with biotin-binding streptavidin. The microbeads were prewashed three times and then resuspended in B & W buffer (5 mM Tris-HCl, 0.5 M EDTA, 1 M NaCl). Then, 10 μL of the buffer, containing 6–7 × 10^5^ microbeads, was mixed with an equal volume of real-time PCR product. The mixture was then incubated at room temperature for 15 min to immobilize the amplicons on the surface of microbeads. The resulting DNA-labeled microbeads were washed three times in deionized water before resuspension in 40 μL of deionized water.

For DEPIM studies of the DNA-labeled microbeads, a chromium castle-walled microelectrode with a gap of 5 μm at its narrowest point, fabricated on a glass substrate, was used, along with an electrical apparatus that was similar to those described previously [5,6]. The DNA-labeled microbead suspension (40 μL) was applied to the microelectrode. The microbeads settled on the microelectrode during 1 min of sedimentation before an AC voltage of 2 V_PP_ and 100 kHz was applied to the microelectrode. The DEPIM conductance curve was then fitted to an exponential function using KaleidaGraph software version 4.1 (Synergy Software, Paramus, NJ, USA).

## 4. Results and Discussion

**Figure 3:**
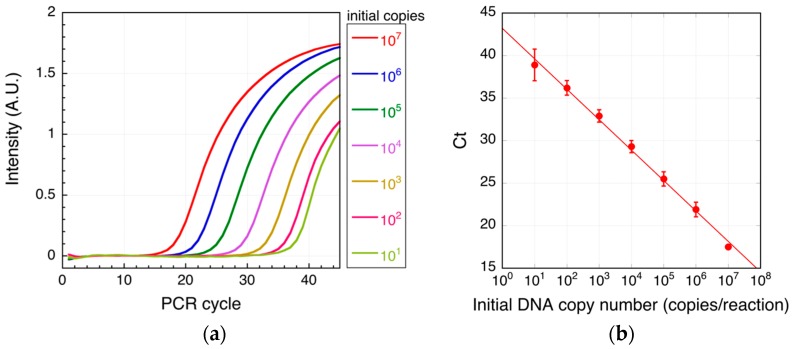
Real-time PCR of target DNA. Shown are typical real-time PCR intensity curves (**a**) and threshold cycles (*C*t; b) for each of the indicated target DNA initial copy numbers. In (**b**), mean values are shown; the error bars are the standard deviation from three independent experiments. The solid line is the fitted line (*R*^2^ = 0.996). *R*^2^ is the coefficient of determination.

First, real-time PCR, with monitoring of the optical intensity after each cycle, was performed using serially diluted target DNA (Figure 3). The threshold cycle (*C*t) at which the amplicon signal was distinguishable from background noise was then plotted for each input DNA copy number, and the effective detection range was determined. We found that target DNA at concentrations of 10–10^7^ copies/reaction could be accurately detected using real-time PCR, with a lower detection limit of 10 copies/reaction (Figure 3b).

**Figure 4:**
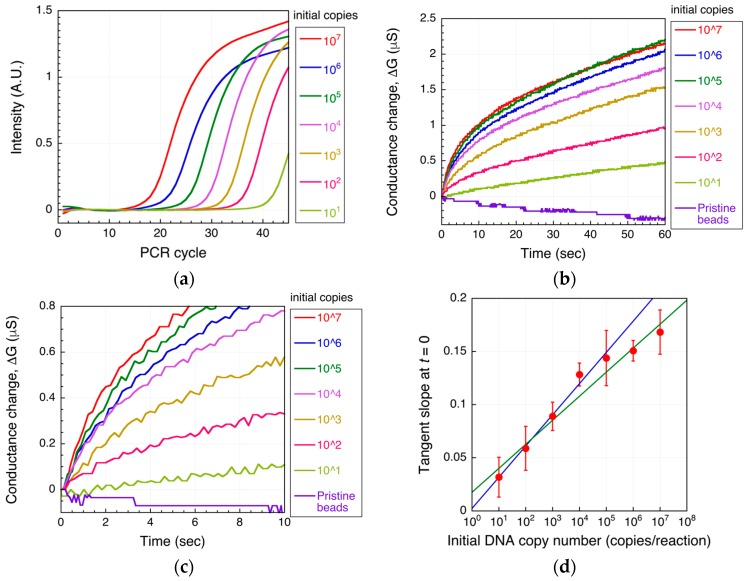
DNA detection using the microbead DEP-based method. Shown are the real-time PCR intensity curve (**a**), the changes in conductance using amplicons of (**a**,**b**), and the initial tangent slope (**d**) for each initial target DNA copy number. A magnification around *t* = 0 of (**b**) is shown in (**c**). In (**d**), mean values are shown; errors bars are the standard deviation from three independent experiments. The blue solid line is the fitted line in the range of 10 to 10^5^ copies (*R*^2^ = 0.988). The green solid line is the fitted line in the range from 10 to 10^7^ copies (*R*^2^ = 0.954).

The biotinylated DNA amplified using real-time PCR at each initial starting copy number was then detected as a conductance change using the microbead DEP-based method described above. The real-time PCR intensity curves and the DEPIM conductance curves are shown in Figure 4a,b, respectively. The conductance of the microelectrode increased with time and with increasing initial target DNA copy number (Figure 4b). The DEPIM curve for 10^6^ copies showed less conductance increase than that for 10^5^ copies because in this case, the amplification from 10^6^ copies reached saturation at an earlier cycle rather than that from for 10^5^ copies (Figure 4a). Additionally, DEPIM curve of 10^7^ copies was similar to that of 10^5^ copies because of saturation (plateau).

In Figure 4d, the tangent slope at *t* = 0 for each DEPIM conductance curve is plotted. In the case of DEPIM for a flowing solution, this slope is proportional to the amount of target present when a constant voltage is applied. In contrast, in a static solution, this slope is proportional to the magnitude of the target DEP force, as described above. In this case, the DNA-labeled microbead DEP force increases with the amplicon amount; thus, the tangent slope also corresponds to the amplicon level. Indeed, Figure 4d shows a linear relationship between the tangent slope and the initial DNA copy number, similar to the case of real-time PCR.

An initial target DNA concentration of 10 to 10^5^ copies/reaction could be effectively quantified using our microbead-based DNA detection method (*R*^2^ = 0.988 for 10 to 10^5^ copies/reaction), whereas initial concentrations of 10^6^ and 10^7^ copies/reaction could not be accurately detected because the number of amplicons produced by real-time PCR reached a plateau, giving a saturated signal (*R*^2^ = 0.954 for 10 to 10^7^ copies/reaction). Therefore, although the lower detection limit of the microbead-based method was equivalent to that of real-time PCR, its dynamic range of detection was narrower. As the method depends on PCR as a first step, this limitation is unavoidable.

It is concluded that the detection limit of the method is similar to that of real-time PCR, and the dynamic range of the method is 10–10^5^ copies/reaction, which is narrower than that of real-time PCR (10–10^8^ copies/reaction).

**Figure 5:**
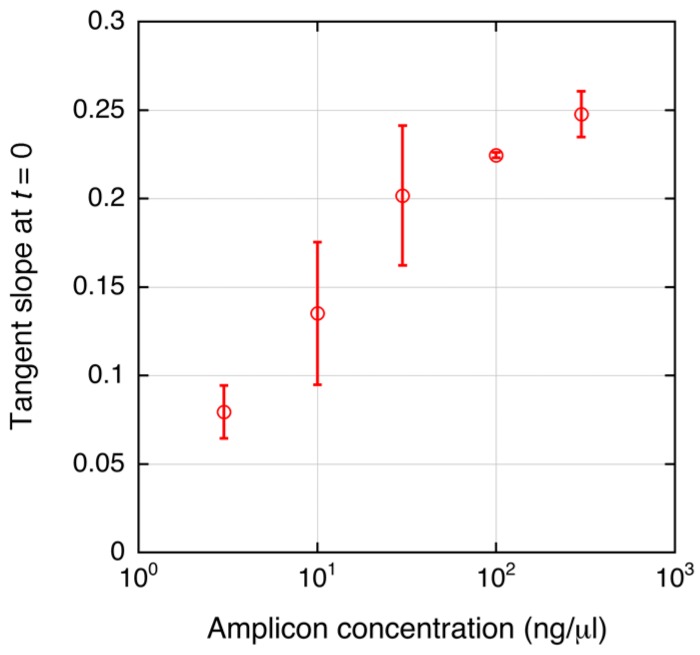
Relationship between amplicon concentration and the tangent slope of the DEPIM curve. PCR-amplified 391-bp DNA was used to evaluate the relationship. Mean values are shown; error bars are the standard deviation from 2 to 4 independent experiments.

**Figure 6:**
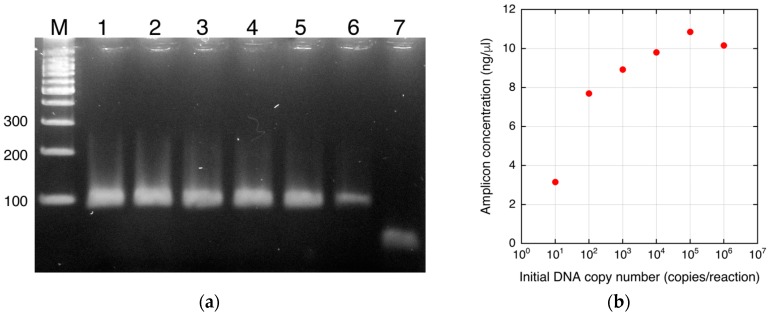
Amplicons of real-time PCR. (**a**) Agarose gel electrophoresis of the amplicons of real-time PCR (Figure 4a,b). M, molecular weight marker; 1–7, amplicons from initial copy numbers of 10^6^–10^0^. (**b**) Plot of the concentration of the amplicons as a function of the initial DNA copy number.

Saturation of the tangent slope corresponding to the DEP force on the DNA-labeled microbeads was observed (Figure 4d). This could have been caused by a plateau of PCR as mentioned above. However, other reasons for the saturation can be considered. The change in the DEP force caused by attachment of the amplicons can also be saturated as shown in Figure 2. To investigate this, the relationship between the DNA amount and the tangent slope was measured as shown in Figure 5. To evaluate the relationship, PCR-amplified 391-bp DNA was used. The DNA was prepared as described in [6]. The slope, corresponding to the magnitude of the DEP force, tended to linearly increase in the investigated range (3–300 ng/μL) in a semi-logarithmic graph. It should be noted that the amount of streptavidin on the microbeads may vary to some extent. However, this variability is acceptable for DNA detection, as shown by the small error bars in Figure 5. Figure 6a shows the agarose gel electrophoresis of the amplicons generated by norovirus real-time PCR (Figure 4a,b). The concentrations of the amplicons estimated using ImageJ are plotted as a function of the initial DNA copy number in Figure 6b. The concentration was estimated by comparing the fluorescence intensity of the band (100 bp) of the size marker, which is known, with that of the band of the amplicons. Note that the small amplicon in lane 7 could be non-specific products. Therefore, the DNA amount of the products in lane 7 was omitted from Figure 6b. The concentration of amplicons tended to be saturated at over 10^5^ copies of the initial DNA. The saturation concentration was approximately 10 ng/μL, whereas the tangent slope as a function of the DNA concentration was not saturated at 300 ng/μL. Therefore, the saturation of the tangent slope shown in Figure 4d can be interpreted as the saturation of the PCR amplification.

A potential limitation of this method is that residual biotinylated primers remain in the PCR solution that is combined with the microbeads, potentially causing aberrant binding effects. However, control experiments showed that the primer alone did not significantly alter the DEP of the microbeads, most likely because the charge of the single-stranded 26-mer primer is negligible compared with that of the approximately 100-bp double-stranded amplicons (data not shown). It is also possible that non-specific products, including primer dimers and other long DNAs, could be amplified by DNA amplification methods similar to PCR. This problem could potentially be minimized by using a sequence-specific attachment scheme, such as previously described hybridization-based DNA-microbead attachment methods [23]. We will examine this in future studies.

There are several methods to detect DNA, such as fluorescence-, electrochemical-, and impedance-based methods. Fluorescence-based methods utilizing miniature real-time PCR have been proposed [24,25]. They require optical setting and fluorescent regents, and are thus costly. There are commercially available portable optical DNA quantification devices, such as Qubit (fluorescence, ThermoFisher Scientific, Waltham, MA, USA) and Nanodrop (fluoerescence/absorptivity, ThermoFisher Scientific), which are used for quantitating the concentration of purified DNA solution. Electrochemical-based detection requires a rather complicated scheme to detect DNA molecules, such as sandwich immobilization on the electrode [26,27,28]. To the best of our knowledge, the detection limit of the electrochemical-based methods is lower than that of our PCR combined method (10 copies/reaction). Electrochemical real-time monitoring methods for amplicons have been also developed [29,30]. These methods can monitor amplicons during PCR without PCR; however, the detection limit is currently not sufficiently sensitive. Surface plasmon resonance-based methods can be performed using a simpler scheme than the electrochemical-based method [31,32]. Recently, a method employing signal enhancement by using a graphene sheet and gold nanoparticles achieved a detection limit of ≈500 aM (≈300 copies/μL) of ssDNA [33]. Impedance-based methods detecting amplicons without oligonucleotide probes such as electrochemical-based methods have been published [34,35]. Although they are simple and easy, there is a risk of false-positive findings because the signal of non-specific products cannot be separated from that of the target in principle. Microbead-based DNA detection methods have been studied [23]. In most of them, DNA attached on the microbeads is detected by fluorescence.

DNA detection using DEP has been reported in several studies [36,37] that employed DEP to collect DNA to a microelectrode. In this case, non-target DNA and protein are also collected by the DEP because their DEP properties are quite similar [38]. In our case, the method consists of PCR and impedance measurement. PCR is used because it provides specific amplification of the target from a very small amount, and modification of the amplicon. Only amplified DNA can be detected because the amplicon has the chemical modification to attach to the microbeads. Moreover, before the impedance detection, the DNA-labeled microbeads are cleaned. Because the microbeads are much larger than impurities such as DNA and protein, the impedance change caused by the impurities should be much smaller than that caused by the microbeads, even though impurities remain in the solution.

Our microbead-based DNA detection method gives a level of quantitation and sensitivity that is equivalent to that of real-time PCR, while also being more cost-effective. Indeed, a DEPIM-based bacteria detection apparatus (Bacterial counter, Panasonic Healthcare, Tokyo, Japan) based on our previous research [39] can be purchased at a lower cost than the standard real-time PCR apparatus. Furthermore, it is our expectation that conventional PCR and microbead-based DNA detection functions can be integrated into one device. Such a device would need to incorporate a buffer exchange system, as DEP is affected by the electrical properties of the surrounding medium, with conductivity in particular being affected by salt concentration. Several types of microfluidic PCR devices have been described previously [24,40].

## 5. Conclusions

In this study, we compared the sensitivity and dynamic range of norovirus genome detection between real-time PCR and a microbead DEP-based DNA detection method. The detection limit of both methods was 10 copies/reaction, although real-time PCR had a larger dynamic range (10–10^7^ copies/reaction) than the DEP-based method (10–10^5^ copies/reaction). The microbead DEP-based method detected amplicons within 20 min. These results suggest that the microbead DEP method, combined with conventional PCR, could offer an alternative to real-time PCR.

## References

[1] Zhao Y., Chen F., Li Q., Wang L., Fan C. (2015). Isothermal Amplification of Nucleic Acids. Chem. Rev..

[2] Mackay I.M., Arden K.E., Nitsche A. (2002). Real-time PCR in virology. Nucleic Acids Res..

[3] Klein D. (2002). Quantification using real-time PCR technology: Applications and limitations. Trends Mol. Med..

[4] Gullett J.C., Nolte F.S. (2015). Quantitative nucleic acid amplification methods for viral infections. Clin. Chem..

[5] Ding Z., Kasahara H., Nakano M., Suehiro J. (2016). Bacterial detection based on polymerase chain reaction and microbead dielectrophoresis characteristics. IET Nanobiotechnol..

[6] Nakano M., Ding Z., Kasahara H., Suehiro J. (2014). Rapid microbead-based DNA detection using dielectrophoresis and impedance measurement. Europhys. Lett..

[7] Suehiro J., Yatsunami R., Hamada R., Hara M. (1999). Quantitative estimation of biological cell concentration suspended in aqueous medium by using dielectrophoretic impedance measurement method. J. Phys. D Appl. Phys..

[8] Nakano M., Hamada R., Takayama H., Shonishi Y., Hisajima T., Mao L., Suehiro J. (2012). Pretreatment of cell membranes for improved electropermeabilization-assisted dielectrophoretic impedance measurement. Sens. Actuators B Chem..

[9] Nakano M., Ding Z., Suehiro J. (2016). Dielectrophoresis and dielectrophoretic impedance detection of adenovirus and rotavirus. Jpn. J. Appl. Phys..

[10] Diehl F., Li M., He Y., Kinzler K.W., Vogelstein B., Dressman D. (2006). BEAMing: Single-molecule PCR on microparticles in water-in-oil emulsions. Nat. Methods.

[11] Viefhues M., Eichhorn R. (2017). DNA dielectrophoresis: Theory and applications a review. Electrophoresis.

[12] Pethig R. (2010). Dielectrophoresis: Status of the theory, technology, and applications. Biomicrofluidics.

[13] Ermolina I., Morgan H. (2005). The electrokinetic properties of latex particles: Comparison of electrophoresis and dielectrophoresis. J. Colloid Interface Sci..

[14] Alberts B.J., Lewis A., Raff J. (2008). Molecular Biology of the Cell.

[15] Bakewell D.J.G., Morgan H. (2004). Quantifying dielectrophoretic collections of sub-micron particles on microelectrodes. Meas. Sci. Technol..

[16] Bakewell D.J., Morgan H. (2001). Measuring the frequency dependent polarizability of colloidal particles from dielectrophoretic collection data. IEEE Trans. Dielectr. Electr. Insul..

[17] Bakewell D.J., Morgan H. (2006). Dielectrophoresis of DNA: Time- and frequency-dependent collections on microelectrodes. IEEE Trans. Nanobiosci..

[18] Loucaides N.G., Ramos A., Georghiou G.E. (2008). Micro- and nano-particle manipulation by dielectrophoresis: Devices for particle trapping and the influence of steric effects. Phys. Status Solidi C.

[19] Loucaides N.G., Ramos A., Georghiou G.E. (2011). Dielectrophoretic and AC electroosmotic trapping of DNA: Numerical simulation incorporating fluid dynamics and steric particle effects. J. Electrostat..

[20] Atmar R.L., Estes M.K. (2001). Diagnosis of noncultivatable gastroenteritis viruses, the human caliciviruses. Clin. Microbiol. Rev..

[21] Fujii M., Yamamoto J., Mukai H., Fujita M., Tsukagoshi H., Yoshizumi M., Saito M., Kozawa K., Kimura H. (2011). Detection and Quantitation of Norovirus Genome Using Real-Time RT-PCR. Jpn. J. Food Microbiol..

[22] Kageyama T., Kojima S., Shinohara M., Uchida K., Fukushi S., Hoshino F.B., Takeda N., Katayama K. (2003). Broadly reactive and highly sensitive assay for Norwalk-like viruses based on real-time quantitative reverse transcription-PCR. J. Clin. Microbiol..

[23] Rödiger S., Liebsch C., Schmidt C., Lehmann W., Resch-Genger U., Schedler U., Schierack P. (2014). Nucleic acid detection based on the use of microbeads: A review. Microchim. Acta.

[24] Ahmad F., Hashsham S.A. (2012). Miniaturized nucleic acid amplification systems for rapid and point-of-care diagnostics: A review. Anal. Chim. Acta.

[25] Ahrberg C.D., Ilic B.R., Manz A., Neužil P. (2016). Handheld real-time PCR device. Lab Chip.

[26] Chao J., Zhu D., Zhang Y., Wang L., Fan C. (2016). DNA nanotechnology-enabled biosensors. Biosens. Bioelectron..

[27] Paleček E., Bartošík M. (2012). Electrochemistry of nucleic acids. Chem. Rev..

[28] Bonanni A., Del Valle M. (2010). Use of nanomaterials for impedimetric DNA sensors: A review. Anal. Chim. Acta.

[29] Teh H.F., Ramalingam N., Dong X.-D., Tan S.N., Zeng X., Kuan A.T.L., Huat E.Y.P., Gong H.-Q. (2009). Real-time PCR microfluidic devices with concurrent electrochemical detection. Biosens. Bioelectron..

[30] Luo X., Xuan F., Hsing I.M. (2011). Real time electrochemical monitoring of PCR amplicons using electroactive hydrolysis probe. Electrochem. Commun..

[31] Šípová H., Homola J. (2013). Surface plasmon resonance sensing of nucleic acids: A review. Anal. Chim. Acta.

[32] D’Agata R., Spoto G. (2013). Surface plasmon resonance imaging for nucleic acid detection. Anal. Bioanal. Chem..

[33] Zagorodko O., Spadavecchia J., Serrano A.Y., Larroulet I., Pesquera A., Zurutuza A., Boukherroub R., Szunerits S. (2014). Highly sensitive detection of DNA hybridization on commercialized graphene-coated surface plasmon resonance interfaces. Anal. Chem..

[34] Salm E., Liu Y.-S., Marchwiany D., Morisette D., He Y., Bhunia A.K., Bashir R. (2011). Electrical detection of dsDNA and polymerase chain reaction amplification. Biomed. Microdevices.

[35] Ma H., Wallbank R.W.R., Chaji R., Li J., Suzuki Y., Jiggins C., Nathan A. (2013). An impedance-based integrated biosensor for suspended DNA characterization. Sci. Rep..

[36] Henning A., Bier F.F., Hölzel R. (2010). Dielectrophoresis of DNA: Quantification by impedance measurements. Biomicrofluidics.

[37] Henning A., Henkel J., Bier F.F., Hölzel R. (2008). Label-free electrical quantification of the dielectrophoretic response of DNA. PMC Biophys..

[38] Zheng L., Brody J.P., Burke P.J. (2004). Electronic manipulation of DNA, proteins, and nanoparticles for potential circuit assembly. Biosens. Bioelectron..

[39] Hamada R., Suehiro J., Nakano M., Kikutani T., Konishi K. (2011). Development of rapid oral bacteria detection apparatus based on dielectrophoretic impedance measurement method. IET Nanobiotechnol..

[40] Yeo L.Y., Chang H.C., Chan P.P.Y., Friend J.R. (2011). Microfluidic devices for bioapplications. Small.

